# Optogenetic techniques for the study of native potassium channels

**DOI:** 10.3389/fnmol.2013.00006

**Published:** 2013-04-11

**Authors:** Guillaume Sandoz, Joshua Levitz

**Affiliations:** ^1^Institute of Biology Valrose, CNRS UMR 7707, INSERM UMR 1091, Université Nice-Sophia AntipolisNice, France; ^2^Institut de Pharmacologie Moléculaire et Cellulaire, CNRS, and Université de Nice Sophia-AntipolisSophia-Antipolis, Valbonne, France; ^3^Laboratories of Excellence, Ion Channel Science and TherapeuticsNice, France; ^4^ Department of Molecular and Cell Biology, Helen Wills Neuroscience Institute, 271 Life Sciences Addition, University of CaliforniaBerkeley, CA, USA; ^5^Biophysics Graduate Group, University of CaliforniaBerkeley, CA, USA

**Keywords:** optogenetic, photoswitchable tethered ligand, photochromic ligand, photoswitchable conditional subunit, K_2P_ channels, TREK-1, SPARK

## Abstract

Optogenetic tools were originally designed to target specific neurons for remote control of their activity by light and have largely been built around opsin-based channels and pumps. These naturally photosensitive opsins are microbial in origin and are unable to mimic the properties of native neuronal receptors and channels. Over the last 8 years, photoswitchable tethered ligands (PTLs) have enabled fast and reversible control of mammalian ion channels, allowing optical control of neuronal activity. One such PTL, maleimide-azobenzene-quaternary ammonium (MAQ), contains a maleimide (M) to tether the molecule to a genetically engineered cysteine, a photoisomerizable azobenzene (A) linker and a pore-blocking quaternary ammonium group (Q). MAQ was originally used to photocontrol SPARK, an engineered light-gated potassium channel derived from Shaker. Potassium channel photoblock by MAQ has recently been extended to a diverse set of mammalian potassium channels including channels in the voltage-gated and K_2P_ families. Photoswitchable potassium channels, which maintain native properties, pave the way for the optical control of specific aspects of neuronal function and for high precision probing of a specific channel’s physiological functions. To extend optical control to natively expressed channels, without overexpression, one possibility is to develop a knock-in mouse in which the wild-type channel gene is replaced by its light-gated version. Alternatively, the recently developed photoswitchable conditional subunit technique provides photocontrol of the channel of interest by molecular replacement of wild-type complexes. Finally, photochromic ligands also allow photocontrol of potassium channels without genetic manipulation using soluble compounds. In this review we discuss different techniques for optical control of native potassium channels and their associated advantages and disadvantages.

## INTRODUCTION

In recent years, the optical control of neuronal activity using genetically encoded actuators, has transformed neuroscience. Up to this point, the major applications of optogenetics have involved the use of light-activated ion channels and pumps to control membrane potential and, thus, action potential firing (Szobota and Isacoff, 2010; Tye and Deisseroth, 2012). This technique has been used most widely for probing synaptic connectivity and the neurological basis of behaviors. However, optogenetics has largely been based on the heterologous expression of opsin-based proteins that are not natively found in the central nervous system. An alternative method using optical control of proteins that are natively expressed in neurons can open the door for a molecular approach to optogenetics. This approach allows light to be used to probe a specific protein from the biophysical to behavioral level with unprecedented precision. A central family of proteins that are ideal candidates for optical control are the many ion channels that lie at the heart of cellular excitability.

**FIGURE 1:**
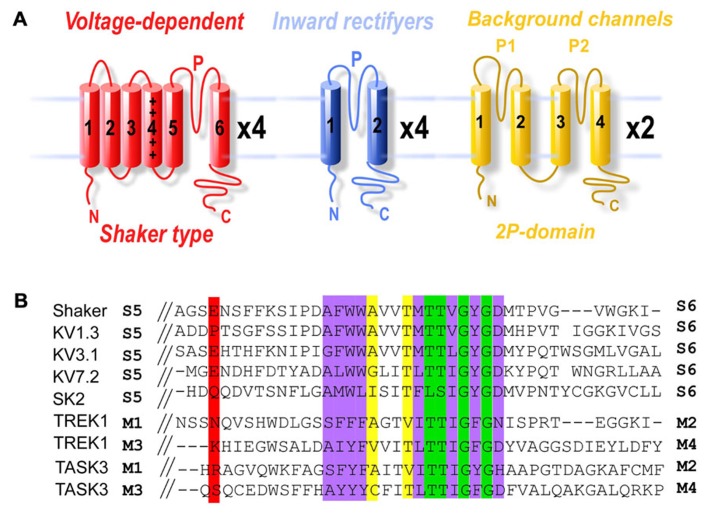
**Domain structure and structural conservation between potassium channel subfamilies.** **(A)** Membrane topology of the three potassium channel families. The shaker family contains six TMs (S1–S6) and one P-domain between S5 and S6. The inward-rectifier potassium channels contain two TMs (S1 and S2) and one P-domain. Finally, the K_2P_ channels contain two pairs of TMs, each flanking a pore domain (in the order: TM1, P1, TM2, TM3, P2, TM4). **(B)** Sequence alignment of potassium channel pore regions. Green boxes indicate positions which have a single, fully conserved residue. Purple boxes indicate conservation between amino acids with strongly similar properties. Yellow boxes indicate conservation between amino acids with weakly similar properties. The red box indicates the E422 cysteine attachment site for Shaker and the equivalent residues in KV1.3, KV3.1, KV7.2, SK2, TREK1 P1 and P2, and TASK3 P1 and P2.

Ion channels generate the electrical signals with which the nervous system senses the world, processes information, creates memories, and controls behavior. The potassium channels represent the most diverse channel subfamily, with 77 genes encoding for different channel subtypes (Coetzee et al., 1999). This diverse set of genes codes for channels with individually unique expression patterns, subcellular targeting, electrical properties, gating, and regulation. Pore-forming subunits share low sequence identity outside the highly conserved pore region called the P-loop or P-domain (**Figure 1**). Four P-loops are necessary to form a functional potassium channel pore. Potassium channels are grouped into three families based on their membrane topology as well as their physiological and pharmacological characteristics. The first family consists of the voltage-gated (Kv) and calcium-gated potassium channels, which includes both the BK and SK channels. These channels consist of four pore-forming subunits which each contain six or seven transmembrane segments (TMs) and one P-loop (**Figure 1A**). The second family is the inward-rectifying potassium channels (Kir) which includes the ATP and G-protein-gated potassium channels. Like voltage-gated potassium channels, Kir channels are also tetrameric and consist of subunits with two TMs and one P-loop (**Figure 1A**). The last family of potassium channels is the two P-domain (K_2P_) potassium channels that produce the leak currents that maintain negative resting membrane potentials. K_2P_ channels, the most recently identified family, consist of a variety of channels with a unique dimeric assembly of subunits containing two P-loops and four TMs (**Figure 1A**; Lesage et al., 1996). The way in which individual members of these three diverse families contribute to the breadth of neuronal function remains a major question in neuroscience. In many cases pharmacological agents that block or activate channels have been used to determine a channel’s function in native systems. However, ligands for blocking specific potassium channels often do not exist. Even when specific ligands do exist they are often selective only at low concentrations which can limit the kinetics and extent of block. Genetic knockout (KO) is an alternative approach to study ion channel function but is also limited due to gene redundancy and potential developmental effects of the KO. Both of these classical techniques make it difficult to determine the role of specific potassium channel subtypes in cellular, circuit, or behavioral properties.

Over the last 8 years, photoswitchable tethered ligands (PTLs) have enabled fast and reversible control of ion channels for both optical control of neuronal activity (Szobota and Isacoff, 2010) and the study of ion channel function (Fortin et al., 2011). PTLs are synthetic molecules that contain a central photoswitchable group, usually azobenzene, connected to a group for tethering to a target protein on one side, and a functional group for manipulating protein activity on the other side. Azobenzene-based PTLs, which toggle between extended *trans* and bent *cis* conformations, provide many advantages for optical control. They are fully reversible, extremely fast (from μs to ms), and often bistable (Beharry and Woolley, 2011). PTLs provide target specificity *via* their covalent attachment to specific proteins with engineered cysteines, even if the functional moiety is a non-specific group. Furthermore, by genetically controlling expression or targeting light, they can allow for spatial control.

Depending on their functional group, PTLs can regulate channel function in two distinct manners in response to specific wavelengths of light that drive a conformational change at the photoswitchable group. PTLs can reversibly present an agonist or antagonist to an allosteric binding site and trigger light-dependent conformational changes that lead to channel activation or deactivation (Volgraf et al., 2006). Alternatively, they can conditionally block the pore of a channel without inducing major conformational changes in the protein (Banghart et al., 2004). The allosteric approach has allowed for the remote control of ligand-gated ion channels including the kainate receptor GluR6 (Volgraf et al., 2006) and the nicotinic acetylcholine receptor (Tochitsky et al., 2012) and G-protein-coupled receptors such as the metabotropic glutamate receptors (Levitz et al., 2013). The conditional block approach has been used to optically control a variety of potassium channels (Fortin et al., 2011). PTLs have been used in a variety of contexts including in cultured cells, intact tissue, and *in vivo* in zebrafish and mice (Wyart et al., 2009; Caporale et al., 2011). This review will focus on PTL-based methods for optical control of potassium channels and recent advances that have extend photocontrol to native proteins.

## OPTICAL CONTROL OF VOLTAGE-GATED POTASSIUM CHANNELS: SPARK AND VARIANTS

**FIGURE 2:**
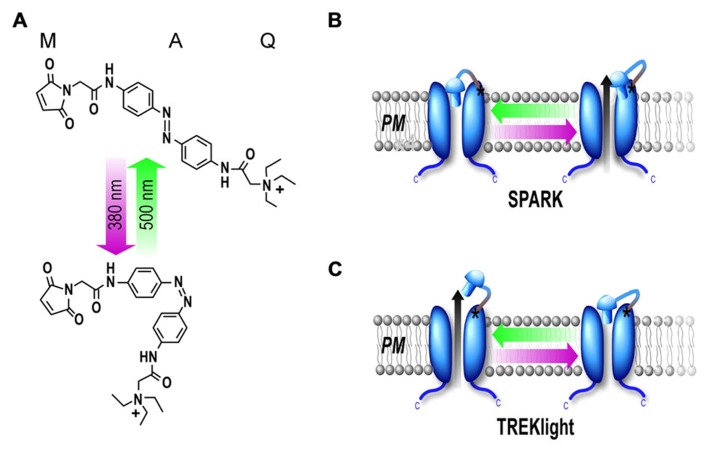
**Photoblock of potassium channels by MAQ.** **(A)** MAQ consists of a maleimide (M), which tethers the photoswitch to a cysteine introduced into the outer portion of the P-loop of the channel, a photoisomerizable azobenzene (A) linker and a quaternary ammonium (Q) pore blocker. In the dark the MAQ is in its relaxed *trans* state but exposure to short wavelength light (380 nm) favors the *cis* state. **(B,C)** Schematic representation of light-gated potassium channels. MAQ is covalently attached to a cysteine outside of the P-loop. MAQ blocks the pore in the *trans *(500 nm light) configuration for SPARK as well as KV1.3, KV3.1, KV7.2, TASK3, and TREK1-K231C. Alternatively, MAQ blocks the pore in the *cis* configuration (380 nm light) for TREKlight (380 nm light) **(C)**.

The first light-gated potassium channel to be developed was SPARK (synthetic photoisomerizable azobenzene-regulated K^+^channel), a modified shaker potassium channel. The Shaker potassium channel was originally cloned from *Drosophila* (Papazian et al., 1987) and has been used as a model protein for the study of voltage-gated ion channels. Voltage-gated potassium channels are blocked extracellularly by the binding of quaternary ammonium ions, such as tetraethylammonium (TEA), to a site in the pore-lining domain (Yellen et al., 1991; Heginbotham and MacKinnon, 1992). It is worth noting that many channels, including potassium, sodium, and calcium channels, also contain an internal TEA binding site. To develop photocontrol of Shaker, the PTL MAQ was developed by Banghart et al. (2004). MAQ contains a maleimide (M) that tethers the molecule to a genetically engineered cysteine, a photoisomerizable azobenzene (A) linker and a pore-blocking quaternary ammonium group (Q). The photoisomerization between *trans* to *cis* azobenzene shortens the molecule from an average of 20 to 13 Å (**Figure 2A**). Shaker residue Glu422, which was estimated to be 15–18 Å from the TEA binding site, was mutated to cysteine to provide an attachment point for MAQ. Conjugation of MAQ to E422C allowed the quaternary ammonium group to block the channel pore when the compound is in the long *trans* form in the relaxed dark state or following visible light (500 nm) illumination. Exposure to short wavelength light, which favors the *cis* form, relieves pore blockage and allows ion conduction. Hence channel conduction can be controlled bidirectionally with light (**Figure 2B** and **Figure 2C**). In addition, this photocontrol is bistable which allows the azobenzene to remain in either *cis* or *trans* following brief illumination with either short or long wavelength, respectively. This property is especially useful for chronic experiments or for combining optical control with optical measurements.

Shaker potassium channels are voltage-gated channels and are not active near the resting membrane potential of neurons (V_1/2_≈-8 mV; Timpe et al., 1988). In addition, they undergo voltage-dependent inactivation (Timpe et al., 1988). Both of these properties make them difficult to use for remote control of neuronal activity. To overcome these obstacles, the authors introduced deletions and mutations to reduce N-type (Δ6-46) and slow inactivation (T449V) of the channel and to shift the voltage-dependence of activation to a more hyperpolarized potential (L366A, V_1/2_≈-36 mV; Lopez et al., 1991). Expression of SPARK in mammalian neurons efficiently reduces action potential firing in response to 380 nm light that is reversed by 500 nm light. To trigger action potentials via 380 nm-induced depolarization in neurons, Chambers et al. (2006) modified SPARK by a single point mutation into the pore-lining domain (Heginbotham et al., 1994), to convert it into a non-selective cation channel termed D-SPARK. When overexpressed in cultured neurons, opening of D-SPARK can trigger light-dependent action potential firing (Chambers et al., 2006). While useful for photocontrol of neuronal activity, SPARK and D-SPARK are non-native and extensively mutated ion channels that do not permit one to study the role of specific potassium channels in neurons.

Because of the high degree of conservation of the pore region of potassium channels, photoblock by MAQ can be generalized to a diverse set of voltage-gated potassium channels, including mammalian isoforms (**Table 1**). Using sequence homology with Shaker, introduction of cysteines into the extracellular loop of mammalian voltage-gated ion channels has been used to endow numerous channels with photosensitivity with similar properties to SPARK (**Figure 1B**). This strategy was applied to three members of different subfamilies of voltage-gated channels including Kv1.3, Kv3.1, and the M-current channel Kv7.2 (**Table 1**). Photocontrol was also applied to one of the Ca^2^^+^-activated K+ channels that generates the long-lasting action potential afterhyperpolarization (SK2; Fortin et al., 2011). Since these channels exhibit different biophysical properties and subcellular targeting, they may enable specific aspects of neuronal function to be controlled by light. Fortin et al. (2011) proposed to use K_V_7.2 to control the resting membrane potential, because it has a low activation threshold and Kv3.1 to control accommodation because these channels have a high-threshold of activation and activate and deactivate rapidly (Gan and Kaczmarek, 1998). Most notably, photoswitchable SK2 channels were used to control the size of EPSPs in CA1 hippocampal neurons where they are natively involved in dendritic repolarization following glutamate receptor-mediated depolarization (Fortin et al., 2011).

**Table 1:** PTL-mediated photoswitchable ion channels.

Channel	Family	Cysteine	*cis* or *trans* block?	Properties	Potential neuronal applications	Reference
*Drosophila*	K_v_	E422C	*trans*	A-type current	Photocontrol of Vm	Banghart et al. (2004)
Shaker				Voltage-gated		
Δ6-46				V_1/2_ = -36 mV		
L366A				Weak inactivation		
T449V						
“SPARK”						
“D-SPARK”	K_v_	E422C	*trans*	Non-selective cation channel	Photocontrol of Vm	Chambers et al. (2006)
V443Q				Voltage-gated		
				V_1/2_ = -36mV		
Kv1.3-H401Y	K_v_1	P374C	*trans*	Voltage-gated	Photocontrol of Accomodation	Fortin et al. (2011)
				V_1/2_ = -30mV		
Kv3.1	K_v_3	E380C	*trans*	Weak inactivation	Photocontrol of Vm	Fortin et al. (2011)
				V_1/2_ = -40mV		
K_v_7.2	K_v_7	E257C	*trans*	M-type current	Photocontrol of Vm	Fortin et al. (2011)
				V_1/2_ = -30mV	Photocontrol of M-current	
SK2	SK	Q339C	*trans*	Ca^2^^+^-activated	Photocontrol of afterhyperpolarization	Fortin et al. (2011)
TREK1/K_2P_2.1	K_2P_	S121C	*cis*	Leak current	Photocontrol of Vm	Sandoz et al. (2012)
“TREKlight”				pH-sensitive		
				Extensive regulation		
TREK1/K_2P_2.1	K_2P_	K231C	*trans*	Leak current	Photocontrol of Vm	Sandoz et al. (2012)
“SRARK-like”				pH-sensitive		
				Extensive regulation		
TREK1 ΔC	K_2P_	S121C	*cis*	Leak current	Photocontrol of native TREK1	Sandoz et al. (2012)
“TREK1-PCS”				pH-sensitive	Conduction	
				Extensive regulation		
TASK3/K_2P_9.1	K_2P_	R73C or A74C	*trans*	Leak current	Photocontrol of Vm	Sandoz et al. (2012)
				pH-sensitive		
				Extensive regulation		

## OPTICAL CONTROL OF K_2P_ POTASSIUM CHANNELS: TREKlight

K_2P_ channels are one of the most diverse and important subfamilies of potassium channels. They serve as a hub for the generation and regulation of a negative resting membrane potential and thus, cellular excitability. In addition to background roles as leak channels, K_2P_channels also play a central role in the dynamic response of cells to extracellular and intracellular signals as diverse as GPCR signaling, pH, and membrane stretch. TWIK-1-related K+ channel 1 (TREK1), a particularly well-studied K_2P_ channel, has been found to be involved in many physiological processes such as neuroprotection against ischemia (Heurteaux et al., 2004), pain perception (Noel et al., 2011), and depression (Heurteaux et al., 2006). Consistent with a proposed role for TREK1 in depression, TREK1 is inhibited by therapeutic doses of selective serotonin reuptake inhibitors (SSRIs) such as fluoxetine (Prozac; Heurteaux et al., 2006; Sandoz et al., 2011) and spadin, a sortilin-derived peptide that also has antidepressive effects (Mazella et al., 2010). Together these properties suggest that TREK1 is an attractive pharmacological target for the development of new antidepressant drugs and provide motivation for a deeper understanding of this channel’s function. However, despite the large interest in understanding the properties of K_2P_ channels, it has been difficult to decipher the precise physiological roles of individual subtypes such as TREK1. For this reason, this channel subfamily is especially attractive for PTL-based optical control.****

K_2P_ channels are believed to be only weakly sensitive or insensitive to extracellular TEA (Noel et al., 2011). However, weakly TEA-sensitive channels may still be sufficiently blocked by MAQ because of the high effective concentration of the tethered quaternary ammonium ligand near the pore in the blocking state. In fact, weakly TEA-sensitive channels may provide a larger difference between the level of block in *cis* and *trans* due to the ease with which QA can be removed from its low-affinity binding site.

As mentioned previously, K_2P_ channels have a unique domain structure relative to all other potassium channels. The domain structure contains four transmembrane helices and two P-loops loops, termed P1 and P2. We first examined cysteine substitutions at residue N122 in P1 and K231 in P2 of TREK1, since these are homologous to the optimal site for photoblock by MAQ in the Shaker channel (Shaker E422) in terms of the number of residues from the selectivity filter (G-X-G motif; Banghart et al., 2004; Sandoz et al., 2012). While both sites showed photo-modulation, they had a different wavelength-dependence. TREK1(K231C-MAQ) produced photoblock in the *trans* state (500 nm illumination), as was also found in Shaker (Banghart et al., 2004) while TREK1(N122C-MAQ) produced photoblock in the *cis* state (380 nm illumination; Sandoz et al., 2012) as was observed for mutation D259C in KV.7.2 (Fortin et al., 2011). This result indicates, surprisingly, that there is some structural asymmetry between the two P-loops in K_2P_ channels. Recent crystal structures have supported this finding (Brohawn et al., 2012, 2013; Miller and Long, 2012).

Further cysteine scanning showed that a single amino acid shift away from N121 TREK1(Q123C), completely eliminates photosensitization (Sandoz et al., 2012). This was also observed for KV7.2 (K255C and G256C) mutations and indicates that photoswitch attachment point is crucial and highly sensitive to the precise residue that is substituted to a cysteine (Fortin et al., 2011). One of the possible explanations for this strict positional requirement is that in many conformations MAQ tethers too far or too close from the quaternary ammonium binding site to enable the quaternary ammonium to reach and bind in the pore. Alternatively, the quaternary ammonium group may be able to block the pore in both *cis* and *trans* conformations, thus preventing a measurable difference between the two states. Finally, at some positions, the genetically engineered cysteine may simply be inaccessible to covalent modification (Fortin et al., 2011). The strongest photoblock observed with TREK1 was measured using the S121C mutation in the P1 domain. Conjugation of MAQ to TREK1-S121C led to up to 70% photoblock of TREK1 current by *cis*-MAQ (380 nm light) that was relieved by *trans*-MAQ (500 nm light). Since MAQ thermally relaxes into the *trans* state, TREK1(S121C-MAQ) (“TREKlight”) has the advantage over previous optogenetic potassium channels that the channel is unblocked and functions normally in the dark for extended time periods and can then be blocked by brief illumination at 380 nm.

To determine if the PTL approach could be further generalized to other weakly TEA-sensitive channels in the K_2P_ family, we extended MAQ photoblock to another K_2P_ channel, TASK3 (Sandoz et al., 2012). TASK3 channels are an attractive pharmacological target since they have been found to be involved in cancer development, inflammation, ischemia, and epilepsy (Bittner et al., 2010). Mutation of residues homologous to TREK N122C and Shaker E422C in TASK3 endows the channel with MAQ-mediated photosensitivity. Like SPARK, TASK3-R73C and A74C undergo sizable photoblock when MAQ is in the *trans* state. Photocontrol of TASK3 indicates that optical control may be extendable within the K_2P_ family and generally, to channels with weak TEA-sensitivity (Sandoz et al., 2012).

When transfected into hippocampal neurons, TREKlight provides a very useful way to remote control membrane potential. TREK1 generates a weakly outward-rectifying leak current which is time and voltage-independent which makes it ideal for modulation of membrane potential since it is always open near the neuronal resting potential. In addition, TREK channel activity can be easily modified by single point mutations in the carboxy-terminal tail that mimic phosphorylated or protonated states (Sandoz et al., 2011). Because of the well-characterized regulatory mechanisms and associated mutants, it is conceivable to produce TREKlight variants with different levels of activity and sensitivity to second messengers (Noel et al., 2011) that may be useful for specific applications in different contexts.

## OPTICAL CONTROL OF NATIVE CHANNELS

**FIGURE 3:**
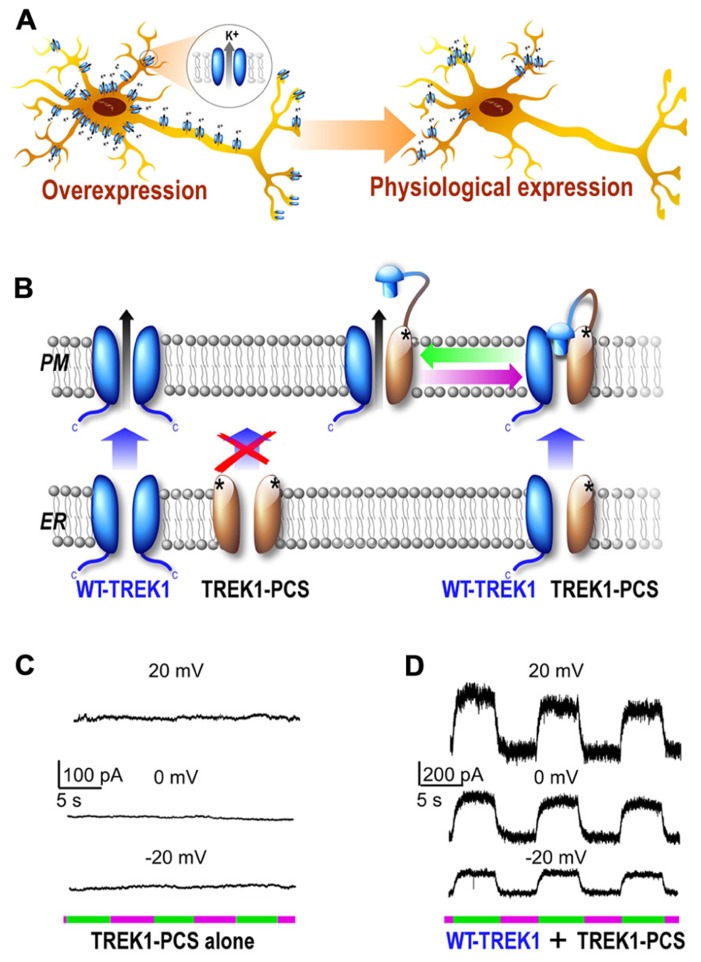
**Development of a subunit replacement strategy for optical control of native potassium channels.** **(A)** Schematic representation of expression of a potassium channel when it is overexpressed compare to its native, physiological expression levels. **(B)** Schematic representation of subunit replacement strategy. Deletion of the TREK1 carboxy-terminal tail (TREK1-PCS, gray) results in retention of the homomeric mutant channel in the endoplasmic reticulum. In contrast, the wild-type homomeric channel (WT, blue) traffics to the plasma membrane. Coexpression of TREK1-PCS with WT produces a heteromeric channel that traffics to the membrane because of the WT subunit and can be light-gated because of MAQ attachment to the TREK1-PCS. **(C,D)** Whole-cell recording from HEK293T cell expressing either TREK1-PCS alone **(C)** or co-expressed with WT **(D)** and labeled with MAQ. Alternating illumination at 500 nm (green) and 380 nm (magenta) reversibly blocks and unblocks constant outward current, as seen at different holding potentials (Sandoz et al., 2012).

While optical control of channels is a powerful way to probe a channel’s structure, gating, and regulatory properties in heterologous systems, in order to delve into a channel’s physiological role one requires a method for manipulation of native channels. Classically, probing of ion channel physiological function requires pharmacological agents or gene invalidation (KO mice) to be addressed. However, as discussed above, selective soluble ligands are lacking for many channels and they lack spatiotemporal precision. Due to the shortcomings of pharmacological tools, gene inactivation has been the principal approach to study the physiological roles of several membrane proteins, including potassium channels. KO mice, which are very useful for a rough characterization of a gene’s function, have many disadvantages for deciphering a precise role for a gene. For instance, gene redundancy can hide the role of the targeted channel. In addition, with a classical KO there is no spatiotemporal resolution of the KO and the absence of the channel during development makes it difficult to decipher whether a phenotype is due to a developmental effect or the channel’s absence in the adult. To overcome these problems, the conditional KO has been developed which permits gene knockout with improved temporal and/or spatial resolution (Friedel et al., 2011). While this technique is a major improvement over the classical KO, temporal control is still slow (on the order of days) and gene knockout is irreversible. A good alternative system would be one in which the function of the protein of interest can be blocked with millisecond precision in a reversible manner, as has been shown with PTLs. However, heterologous expression of a photoswitchable channel leads to a greater channel density than the endogenous channel. In addition, overexpressed channels may not target specifically the way native channels do (Figure 3A).

## PHOTOCHROMIC LIGANDS

One approach that has been used to photocontrol native channels has been the so-called photochromic ligands (PCL; Volgraf et al., 2007; Kramer et al., 2009). These compounds, like PTLs, consist of an azobenzene coupled to a functional moiety. However, instead of a tethering group, a chemically inert or electrophilic group, such as acrylamide (AAQ) or epoxide (EAQ), is added to the opposite side of the azobenzene. PCLs based on quaternary ammonium ligands have been shown to successfully photocontrol a variety of ion channels. Initially, these compounds were believed to covalently attach to a native nucleophilic group on voltage-gated potassium channels and to photosensitize channels via the external TEA site (Fortin et al., 2008). However, subsequent work indicated that PCLs enter the cell and photosensitize a wide range of voltage-gated channels via the internal TEA site (Banghart et al., 2009; Fehrentz et al., 2012). Most notably, such compounds have been used for photocontrol of ion channels in the retina for vision restoration in blind mice (Tochitsky et al., 2012) and for optically controlled analgesia via a PCL called quaternary ammonium-azobenzene-quaternary ammonium (QAQ) that specifically enters nociceptive ion channel-expressing cells (Mourot et al., 2012). However, despite their power for control of membrane potential, in most cases PCLs lack target specificity which makes it difficult to determine a particular channel’s contribution. In the future PCLs with specific ligands for potassium channels, as has recently been done for α-amino-3-hydroxy-5-methyl-4-isoxazolepropionic acid (AMPA) receptors (Stawski et al., 2012), may be developed. Furthermore, PCLs are also more difficult than PTLs to target spatially because they are not used in conjunction with genetic control and are subject to diffusion.

## CONTROL OF ENDOGENOUS CHANNELS VIA A KNOCK-IN MOUSE

Another option for the optical control of potassium channels without overexpression is to introduce the cysteine mutation for MAQ conjugation into the native protein via genetic knock-in. This technique will produce a mouse in which the wild-type channel (WT) gene is replaced by its photoswitchable version in the exact same place in the genome, which should preserve expression pattern and levels. Since, in most cases tested so far, light-gated channels have the same properties as WT channels (regulation, conductance, etc.) the only phenotypic difference should be the ability to control the channel with light. A knock-in mouse would allow one to address the channel’s function *ex vivo* and, potentially, *in vivo* without exogenous gene delivery. All that is required for such experiments is to conjugate MAQ, apply light and measure the difference in some measurable quantity, such as current or a behavior, before and after applying 380 or 500 nm light. Future work will have to determine if this technique is applicable *in vivo* where stereotaxic MAQ injection and light delivery with optical fibers may be employed. However, despite many technical advances, the generation of a knock-in animal is laborious, expensive, and takes at least 1 year. In addition, a knock-in mouse does not allow one to easily separate the contributions of different populations of a given channel in different cell types because all cell types that natively express the channel will contain the cysteine-substituted version. This is relevant in many cases where the same channel is expressed in excitatory and inhibitory neurons, as well as astrocytes in the same region.

## PHOTOSWITCHABLE CONDITIONAL SUBUNITS

Due to the aforementioned shortcomings of existing techniques, we have developed a novel scheme for specific optical remote control of native proteins using a “photoswitchable conditional subunit (PCS).” The PCS approach allows genetic techniques to be used to target expression while maintaining native expression levels and patterns (**Figure 3A**). This approach is generally applicable to multimeric membrane proteins, including channels, and requires two conditions to be met.

First, the channel of interest must be made photoswitchable through a mutation to anchor the PTL. It may be challenging to determine the location of the engineered cysteine depending on the structural data available and the homology to previous photosensitized channels. Furthermore, this cysteine mutation and the subsequent tethering of the photo-activatable reagent may not always be orthogonal and could affect some aspect of channel function. Therefore, functionality of such proteins must always be tested. Importantly, the channel, though multimeric, must be photoswitchable with a PTL attached only to one subunit. In the case of MAQ-mediated photocontrol this is feasible since only one quaternary ammonium moiety may block the channel at a time, anyway (Fortin et al., 2011).

Second, a mutation (or deletion) that prevents the channel to reach the plasma membrane must be added to the photoswitchable subunit. The subunit containing a mutation for PTL anchoring and another mutation for intracellular retention is termed the PCS. In cells lacking native subunits, the PCS remains non-functional and inside of the endoplasmic reticulum (ER). However, in cells that express native subunits, the WT subunit and PCS subunit co-assemble in the ER and the WT subunit facilitates trafficking of the heteromeric complex to the plasma membrane. Once at the plasma membrane and labeled with a PTL, the PCS allows native channels to be controlled with light (**Figure 3B**). It is important to note that this technique requires that the wild-type subunit is able to rescue the trafficking-impaired PCS. In addition, the deletion or mutation of the exporting site must be inert with regard to protein function. Function and regulation of the WT-PCS heteromer must be tested to assure that the general behavior of the complex reflects the native WT complex Furthermore, it is important to note that precise expression levels may not be perfectly maintained. The large pool of PCS subunits may induce an increase in expression level relative to native levels by maximum doubling the number of subunits available for trafficking to the plasma membrane. However, the PCS method is likely to maintain expression levels much closer to physiological levels compared to classical overexpression. Finally, in native systems the replacement of WT complexes with PCS-WT heteromers will be temporally limited by the rate of channel turnover and may lead to incomplete wild-type channel replacement.

The PCS technique was successfully applied to TREK1 and was based on the previously described TREKlight (Sandoz et al., 2012). To develop the TREKlight PCS, the TREK1 carboxy-terminal tail was deleted (TREK1ΔC) which resulted in the retention of the channel in the endoplasmic reticulum as previously described (Chemin et al., 2005). Consistent with this, expression of TREK1ΔC-S121C (“TREK1-PCS”) yielded no potassium current and no detectable photocurrent in HEK 293 cells following MAQ conjugation (**Figure 3C**). In contrast, coexpression of TREK1-PCS with WT-TREK1 yielded a photoswitchable TREK1 current. This result indicated that the TREK1-PCS co-assembles with WT-TREK1 subunits and that the heteromeric channel (TREK1-PCS/WT-TREK1) goes to the cell surface where it is regulated by light via photoisomerization of MAQ attached to the TREK1-PCS subunit. Importantly, the TREK1-PCS/WT-TREK1 heterodimer maintained wild-type internal and external regulation and rectification properties. Furthermore, TREK1-PCS transfection in native tissue allowed the replacement of the WT-TREK1 dimer by the TREK1-PCS/WT-TREK1 heterodimer. In cultured hippocampal neurons and hippocampal slices, the PCS was used to show that TREK1 contributes to a leak current that contributes to the maintenance of the negative resting potential. Unexpectedly this technique also revealed that in addition to its classical role as a leak channel, TREK1 contributes to the hippocampal GABA_B_ response. This result breaks with the traditional notion that Kir3 channels are the sole targets of postsynaptic GABA_B_ receptors and would have been difficult to measure without the PCS technique. In the future, the genetic and optical control of native TREK1 afforded by the TREK1-PCS may allow the determination of the spatiotemporal properties and physiological significance of GABA_B_ activation of TREK channels in the hippocampus.

As demonstrated with TREK1, the PCS method is a powerful way to remote control the activity of native ion channels with subtype specificity. This strategy can be extended to a wide variety of other membrane protein complexes including both channels and receptors. The major limiting factors to applying the PCS to a given membrane protein are the ability to endow the proteins with photosensitivity and to find a mutation or variant with impaired trafficking that may be rescued by the WT version. However, the number of photoswitchable proteins is rapidly increasing and for many membrane proteins trafficking mutants have been identified. For example, forward trafficking signals which may be mutated to prevent trafficking to the plasma membrane which can be rescued by WT subunits have been described for several proteins including the potassium channels Kir1.1 (Heusser et al., 2002), Kir2.1 (Ma et al., 2001), Kir3.2 (Ma and Jan, 2002), Kir3.4 (Ma and Jan, 2002), TASK1 (Girard et al., 2002), and TASK3 (Zuzarte et al., 2007). Interestingly, the strategy to rescue trafficking of one subunit by another is commonly used in nature to ensure that only heteromeric assemblies of a particular protein reach the cell surface. This has been observed for Kir6 channels, where the channel-forming subunit is retained inside the cell unless it is co-assembled with SUR (Sakura et al., 1995; Zerangue et al., 1999). It is also seen with the GABA_B_ receptor, the nicotinic acetylcholine receptor, kainate receptors, and the N-methyl-D-aspartate (NMDA) receptor (Okabe et al., 1999; Standley et al., 2000; Xia et al., 2001). In these cases of obligatory heteromerization, the only condition required to use the PCS approach is to endow a subunit with photosensitivity that is able to regulate the entire protein complex. Overall, the large body of biophysical, structural, and expression/trafficking information that has been attained for potassium channels and other membrane protein complexes should facilitate their application to the PCS method.

## CONCLUSION

In recent years optogenetics has emerged as a transformative field that is based on a combination of genetic and optical tools which can be used to control neuronal activity with high spatiotemporal precision. The majority of optogenetic studies have used opsin-based proteins such as channelrhodopsin-2 (ChR2), a non-selective light-activated cation channel that can depolarize neurons or halorhodopsin (Halo or NpHR), a light-driven Cl^-^ pump that can hyperpolarize neurons (Tye and Deisseroth, 2012). Opsins possess a number of advantageous properties for optogenetics such as their intrinsic photosensitivity which does not require addition of a chemical photoswitch. Their ability to be easily genetically targeted and easily activated *in vivo* has led to a number of elegant studies probing neural circuit function and the neural basis of behavior. However, opsins are unable to mimic the precise properties of natively expressed proteins and are ill-suited for many molecular studies of neuronal function.

An alternative to opsins which allows one to use the advantageous properties of optical control to photocontrol natively expressed proteins is to use PTLs. PTLs have enabled fast and reversible control of a number of ion channels such as SPARK, LiGluR, and HyLighter. These modified channels have been shown to contain a diverse and complementary set of advantages compared to opsins such as thermal bistability and a larger conductance. However, they require the addition of the synthetic PTL to introduce light sensitivity which may complicate experiments (Szobota and Isacoff, 2010). Within the potassium channel family the PTL MAQ has been used to photocontrol two of the three major families of mammalian potassium channels including both the voltage-gated potassium channels and the two-pore domain, K_2P_, channels (**Table 1**). Future work will determine if the third family, Kir, is amenable to MAQ-mediated photoblock. Beyond TEA-sensitive potassium channels, the modular design of PTLs should allow other pore blockers to be tethered to the azobenzene where necessary.

While many of the photoswitchable channels described are useful for remote control of neurons, they may also be used to attain a deeper, molecular understanding of the roles of individual channels in neuronal behavior. Since these modified channels (often a single point mutation) are based on channels that are natively expressed in mammalian neurons and behave like the WT versions on which they are based, the superior specificity and spatiotemporal precision which they offer make them ideal for probing the molecular physiology of the channel itself. Since overexpression is often not suitable for the study of a channel’s function, it has been proposed to use the knock-in strategy to allow for control of endogenous channels (Fortin et al., 2011). However, as a cheaper, faster, and more flexible alternative to KI mice, we have described the PCS approach which was initially applied to the K_2P_ channel, TREK1. The PCS technique offers an affordable, fast and powerful strategy for optical control of native channels which may be used to identify the molecular basis of unknown ionic currents that have been difficult to probe with pharmacological or genetic tools. For example, the TREK1-PCS was used to identify TREK1 as a target of the GABA_B_ receptor activation-induced current in the hippocampus (Sandoz et al., 2012). The PCS approach has a general design that may be applied to many potassium channels and other membrane proteins to probe their physiological roles with superior precision compared to classical techniques. Importantly, in any application of the PCS technique a number of challenges must be overcome. These challenges include developing photocontrol via a PTL, controlling trafficking in a way that may be rescued by the WT subunit, and maintaining wild-type function in a wild-type-PCS heteromer. While each aspect of PCS development may be difficult it is important to note that homology with currently existing photoswitchable proteins, the existence of native mechanisms for control of membrane protein trafficking, and the large body of structural and functional data on channels and receptors should help facilitate such work.

Finally, future engineering efforts may be used to expand the optical properties of the photoswitches used for PTL-mediated photocontrol. Recent work red-shifting the soluble PCL compounds (Mourot et al., 2011) indicates that red-shifted PTLs with altered relaxation rates may be produced which offer advantageous properties, including superior tissue penetration, which may be useful in different contexts.

## Conflict of Interest Statement

The authors declare that the research was conducted in the absence of any commercial or financial relationships that could be construed as a potential conflict of interest.
